# Forestwalk: A Machine Learning Workflow Brings New Insights Into Posture and Balance in Rodent Beam Walking

**DOI:** 10.1111/ejn.70033

**Published:** 2025-03-11

**Authors:** Francesca Tozzi, Yan‐Ping Zhang, Ramanathan Narayanan, Damian Roqueiro, Eoin C. O'Connor

**Affiliations:** ^1^ Neuroscience and Rare Diseases Discovery and Translational Area, Roche Pharma Research and Early Development, Roche Innovation Center Basel F. Hoffmann‐La Roche Ltd Basel Switzerland; ^2^ Data and Analytics, Roche Pharma Research and Early Development, Roche Innovation Center Basel F. Hoffmann‐La Roche Ltd Basel Switzerland

**Keywords:** Angelman syndrome, balance, beam walk, mouse, SLC6A1‐related neurodevelopmental disorder

## Abstract

The beam walk is widely used to study coordination and balance in rodents. While the task has ethological validity, the main endpoints of “foot slip counts” and “time to cross” are prone to human‐rater variability and offer limited sensitivity and specificity. We asked if machine learning–based methods could reveal previously hidden, but biologically relevant, insights from the task. Marker‐less pose estimation, using DeepLabCut, was deployed to label 13 anatomical key points on mice traversing the beam. Next, we automated classical endpoint detection, including foot slips, with high recall (> 90%) and precision (> 80%). Using data derived from key point tracking, a total of 395 features were engineered and a random forest classifier deployed that, together with skeletal visualizations, could test for group differences and identify determinant features. This workflow, named Forestwalk, uncovered pharmacological treatment effects in C57BL/6J mice, revealed phenotypes in transgenic mice used to study Angelman syndrome and SLC6A1‐related neurodevelopmental disorder, and will facilitate a deeper understanding of how the brain controls balance in health and disease.

## Graphical abstract

Forestwalk is an advanced analysis pipeline for the beam walking test in mice, which automates the detection of classical endpoints (i.e., number of foot slips and time to cross the beam) and delivers more sensitive and specific insights into postural control and balance.

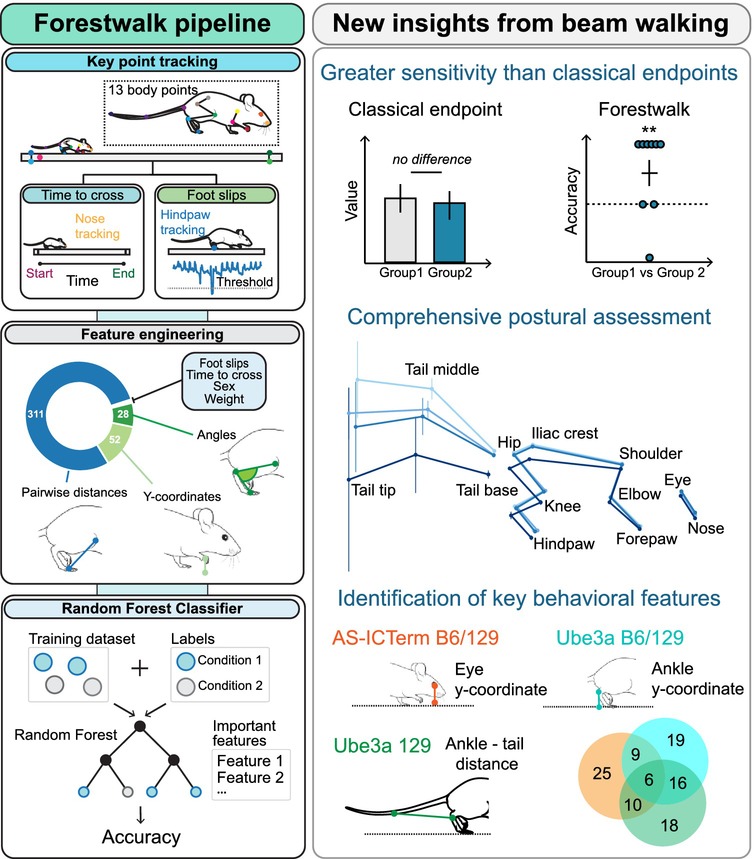

**Abbreviations**
AAALACi: Association for Assessment and Accreditation of Laboratory Animal Care International
ANCOVA: analysis of covariance
ANOVA: analysis of variance
AS: Angelman syndrome
DLC: DeepLabCut
EEG: electroencephalography
FN: false negative
FP: false positive
GAT1: gamma‐aminobutyric acid transporter 1
HE: heterozygous
HO: homozygous
ID: identification
KNN: k‐nearest neighbors
KO: knockout
LDA: linear discriminant analysis
LOMO‐CV: leave‐one‐mouse‐out crossvalidation
MMRRC: Mutant Mouse Resource and Research Center
NB: naive Bayes
NIH: National Institutes of Health
RFC: random forest classifier
RFE: recursive feature elimination
SFMA: single‐frame motion analysis
SHIRPA: SmithKline, Harwell, Imperial College, Royal Hospital, Phenotype Assessment
SimBA: simple behavioral analysis
SVM: support vector machine
TP: true positive
WT: wild‐type

## 1 Introduction

A wide variety of tests have been developed to measure motor function in rodents (Brooks and Dunnett 2009). These include measures of gross neurological function, such as the SmithKline, Harwell, Imperial College, Royal Hospital, Phenotype Assessment (SHIRPA; Rogers et al. 2001), to others that provide insights into fine motor skill and learning, such as directional reaching tasks (Galiñanes et al. 2018). Motor coordination and balance are typically measured with either a rotarod, or in climbing and swimming tasks, or in beam walking and stepping tests (Brooks and Dunnett 2009; Carter et al. 2001). The beam walk was originally established to detect damage to the motor cortex in rats and mice (Feeney et al. 1982; Gentile et al. 1978; Goldstein and Davis 1990; Piot‐Grosjean et al. 2001) but has since been deployed across multiple disciplines and disease areas, including for drug pharmacology and toxicology (Karl et al. 2003; Stanley et al. 2005), the study of aging (Gage et al. 1984; Wallace et al. 1980), spinal cord injury (Ito et al. 2022; Semler et al. 2011), neuromuscular disorders (Liu et al. 2022), stroke (Alexis et al. 1995), Alzheimer's disease (Montbrun et al. 2019), Parkinson's disease (Bidgood et al. 2024), Huntington's disease (Carter et al. 1999), and multiple sclerosis (Sen et al. 2020). Of note, beam walking is also used to study balance and coordination in humans (Chaumeil et al. 2024; Sawers and Ting 2015) and thus holds additional value to enable direct comparison of test performance across species.

In the beam walk, rodents are required to traverse a suspended narrow beam to reach a goal area (Brooks and Dunnett 2009; Carter et al. 2001; Modi et al. 2024). The length, shape, diameter, material, and angle of the beam can be varied in order to change the complexity of the task and reveal deficits in coordination and balance that may otherwise not be evident from kinematic observations made on a flat walking surface (Lang et al. 2020). The popularity of the test likely resides in the fact that it is simple to set up, is inexpensive, and can be performed after limited training and with minimal expertise of the experimenter (Carter et al. 2001). The two primary endpoints typically reported in the beam walk are “the number of foot slips of the hind paw” and “the time to cross the beam” (Bidgood et al. 2024; Brooks and Dunnett 2009; Carter et al. 2001; Hausser et al. 2018; Modi et al. 2024). Additional measures such as number of falls or total distance traveled on the beam may also be reported but are typically only changed in models with more severe phenotypes (e.g., with spinal cord injury [Carter et al. 2001]).

Although widely used and easy to implement, a major limitation of the beam walk test is that its primary endpoints, including the number of foot slips and time to cross, are typically scored by human raters. Human raters are well known to demonstrate high levels of inter‐ and intra‐rater variability and likely contribute to reduced reliability and reproducibility of findings. Human‐rater scoring is also very time intensive (Carter et al. 2001) and is only possible with a limited number of observations that are readily identified by the human eye. Consequently, subtle changes in behavior that are challenging to quantify can escape entirely from human detection (A. Mathis et al. 2020; M. Mathis and Mathis 2020; Nilsson et al. 2020; von Ziegler et al. 2021). Together with the limited *sensitivity* of endpoints available to human raters, a restricted set of endpoints also leads to poor *specificity* of the beam walk test to discriminate between fundamentally different experimental conditions. Numerous studies report increases in foot slips in beam walking, despite using rodent models with nonoverlapping disease pathomechanisms. Taken together, achieving automated endpoint detection in the beam walk could lead to more robust and reproducible experimental findings. Furthermore, increasing both the sensitivity and specificity of beam walk endpoints to detect and discriminate subtle yet relevant functional changes is of critical importance if the test is to reliably inform on the link between brain function and posture and balance control.

Motivated by these opportunities, we set out to use modern computational neuroethology methods to gain greater insight into posture and balance in beam walking in mice (Luxem et al. 2022; A. Mathis et al. 2018; Nilsson et al. 2020; von Ziegler et al. 2023; Weinreb et al. 2024; Wiltschko et al. 2020). While other reports have also proposed alternative data capture and analysis methods for beam walking, they either still rely on human‐rater scoring and annotation (Apostolova et al. 2006; Irintchev et al. 2005; Ito et al. 2022; Semler et al. 2011), or are unable to automate detection of foot slips (Bidgood et al. 2024; Lang et al. 2020), or have not demonstrated the scalability of the proposed approach to full experimental conditions (Wan et al. 2023). To address these gaps, we first deployed DeepLabCut (DLC), an open‐source toolbox that allows training of a deep neural network to annotate animal key points to human‐level accuracy (A. Mathis et al. 2018). Using DLC key points from each mouse recorded during beam walking, we subsequently developed an analysis workflow that fully automated the detection of the classical endpoints of the number of foot slips and time to cross the beam. A total of 395 features were then engineered from each beam walk test and used to train a random forest classifier (RFC) to detect differences between experimental groups and reveal determinant features that, together with skeletal representations of mice, could provide greater insight into posture and balance than achieved by classical endpoints alone. We show that the feature set engineered specifically for Forestwalk outperforms generic feature sets provided by a popular tool used to develop supervised behavioral classifiers (i.e., simple behavioral analysis, SimBA [Goodwin et al. 2024]). Proof of principle for this workflow, named Forestwalk, is given by increased sensitivity to detect pharmacological effects of diazepam and the ability to identify similarities and differences among transgenic mouse strains commonly used in the study of two neurodevelopmental disorders, namely, Angelman syndrome (AS) and SLC6A1‐related neurodevelopmental disorder. Taken together, Forestwalk is an efficient, automated, and open‐source workflow that scales to whole experiments and represents the current state of the art for advanced analysis of beam walking in mice.

## 2 Materials and Methods

### 2.1 Animals

For pharmacology studies with diazepam, male C57BL/6J mice (Jax ID: 000664, aged 14 weeks old, *n* = 8 mice) were obtained from Charles River Laboratories (Saint Germain sur l'Arbresle, France). Experiments in genetic models for neurodevelopmental disorders included male and female, mutant and wild‐type (WT) littermates of Ube3a 129 mice (129‐Ube3a^tm1Alb^/J, Jax ID: 004477; aged 8 weeks, *n* = 16 WT mice, *n* = 16 knockout [KO] mice); Ube3a B6/129 hybrid mice (B6.129S7‐Ube3a^tm1Alb^/J, Jax ID: 016590; aged 9 weeks, *n* = 16 WT mice, *n* = 16 KO mice); AS‐ICTerm mice (C57BL/6J‐Rr70^em1Rsnk^/Mmmh, 065423‐MU; aged 7–8 weeks, *n* = 16 WT mice, *n* = 16 KO mice); and GAT1 mice (B6.129S1‐Slc6a1^tm1Lst^/Mmucd; 000426‐UCD; aged 18 weeks, *n* = 18 WT mice, *n* = 20 HE mice, *n* = 10 HO mice). All transgenic mice were bred externally (Taconic, Denmark or Charles River, Germany) and shipped to the test facility (Roche Innovation Center, Basel) after weaning. Mice were acclimated to the test facility for at least 1 week before starting experiments. All animals were experimentally naive prior to starting beam walk experiments.

Mice were housed in groups of 2–3 per cage (GM500, Tecniplast), unless instances of aggression between littermates required single housing. Woodchip bedding (SAFE FS 14, J. Rettenmaier & Söhne GmbH) was used in cages, with nesting material and additional in‐cage enrichment items provided, which were replaced during each cage change. Mice had ad libitum access to food (Standard Diet; Kliba Nafag) and water in the home cage. Temperature (22°C ± 2°C) and humidity (50 ± 10%) were controlled in housing and experimental rooms, and mice were maintained under a 12 h:12 h Light:Dark cycle (lights transitioning to off at 6:00 p.m.), with beam walk tests conducted during the light phase.

All animal experiments were conducted in strict adherence to the Swiss federal ordinance on animal protection and welfare, as well as according to the rules of the Association for Assessment and Accreditation of Laboratory Animal Care International (AAALACi), and with the explicit approval of the local veterinary authorities.

### 2.2 Beam Walk Test

The beam walk test was employed to evaluate rodent coordination, posture, and balance (Brooks and Dunnett 2009; Luong et al. 2011). This assessment measures the ability of the rodents to traverse a series of 1‐m long narrow beams of varying diameters and shapes (16 mm square, 16 mm round, 9 mm square) to reach a goal area (a dark box containing a home cage with clean bedding material). The beams were suspended 30 cm above a bench, with a soft pad placed underneath to cushion any falls. Prior to the beam walk test, mice underwent a minimum of three training trials to ensure their ability to cross the beam. These trials progressed from first placing the mouse in close proximity to the box, to the middle of the beam, and finally at the start of the beam. Before the start of the test, mice were acclimated for 2 min in the goal area. In a standard experiment, mice were required to traverse the 16‐mm square rod twice (i.e., Trials 1 and 2), followed by the 16‐mm round rod twice, and finally the 9‐mm square rod twice. A rest period of 15 min was provided between each rod. In pharmacological experiments, the protocol was slightly modified. Mice traversed each beam once (i.e., Trial 1 on the 16‐mm square rod, followed by Trial 1 on the 16‐mm round rod, and then by Trial 1 on the 9‐mm square rod). Following completion of this sequence, mice were rested for a minimum of 15 min. The test sequence was then repeated to obtain data for Trial 2 on each rod. This configuration facilitated a more comparable assessment of diazepam's effect across the three different beam types. In instances where a mouse fell from the beam, a second attempt on the same trial was permitted. However, a second fall resulted in the termination of all subsequent beam walk testing for that mouse on that day. When a mouse fell from the beam during a given trial, data from the partially completed trial were not included in the analysis.

### 2.3 Pharmacological Treatment

On the test day, mice (*n* = 2 per dose treatment group; total study *n* = 8) were pseudorandomly assigned to receive either diazepam (synthesized by F. Hoffmann‐La Roche) (0.3, 1, or 3 mg/kg) or its vehicle (NaCl 0.9% + Tween 80 0.3%) administered intraperitoneally 30 min before starting test trials. The experiment was repeated once per week until all animals received all treatment conditions, following a within‐subjects–randomized crossover design.

### 2.4 Pose Estimation and Tracking

Behavioral videos were acquired using a Basler C‐Mount acA1920‐150um camera (2.3 MP resolution, 120 fps; Basler, Germany) and a HF25XA‐5M. F1.6/25‐mm lens (Chromos Group AG, Switzerland). DeepLabCut 2.2.3 was used to track 18 key points, including 5 reference points on the beam and 13 mouse body parts including the nose, the eye, the forepaw, the elbow, the shoulder, the hindpaw, the ankle, the knee, the hip, the iliac crest, the base of the tail, the tail center, and the tail tip. The final network was trained by using a total of 520 frames extracted from 26 videos for 1,030,000 iterations.

### 2.5 Forestwalk Workflow

The coordinates extracted by DLC were processed by a custom‐made Python script, and the points marked on the beam were used to perform the automatic detection of the beam type (16‐mm square beam: a small dot on the right side; 16‐mm round beam: no dot; 9‐mm square beam: small dot on the left side). The central 80‐cm region of the beam was detected using the key points tracking the two vertical black lines displayed at the two extremities of each beam. The final error of the network was 3.11 pixels on the training set and 3.82 pixels on the test set, with the training set representing 95% of the data.

#### 2.5.1 Automated Detection of Time to Cross and Number of Foot Slips

The method developed for automated detection of time to cross and number of foot slips is given in Section 3.1. Foot slip detection accuracy was evaluated by comparing an automated threshold–based method with reference annotations made by a single human scorer. A true positive (TP) was identified when the automated method's detection aligned with a foot slip noted by the scorer within a predetermined frame range. A false negative (FN) occurred when the automated method did not detect a slip that was identified by the scorer, while a false positive (FP) was a detection by the automated method not confirmed by the scorer. Various thresholds were tested to fine‐tune the automated method. Performance metrics—recall (TP / (TP + FN)), precision (TP / (TP + FP)), and the F1 score—guided the selection of the optimal threshold, which was determined by the highest combined recall and precision to ensure a balanced detection of slips with minimal FP and FN identifications. Agreement between different human scores and the automated method for foot slip detection was calculated using Cohen's kappa. Cohen's kappa coefficient was calculated using the “cohen_kappa_score” function from Sklearn.metrics (scikit‐learn library; Pedregosa et al. 2011). Agreement was assessed on a per‐video basis by comparing the total foot slip counts identified by each scorer for each video, providing a video‐level measure of scorer consistency.

#### 2.5.2 Custom Feature Engineering for Forestwalk

Pose estimation data collected from each behavioral video were utilized to generate a set of behavioral features. Features were obtained by calculating all the pairwise distances between the animal's body parts throughout the video, the angles at the elbow, ankle, knee, and hip levels, and the distance of each body part from the beam surface. These features were selected to provide a detailed representation of the animal's posture and movement dynamics that we believed would be of most relevance to the task. Specifically, the inclusion of pairwise distances and key angles helps to reveal spatial relationships and joint configurations, which are critical for understanding postural control and coordination. Likewise, the height of body parts from the beam surface reflects vertical positioning and potential deviations that can implicate balance. To account for differences in animal body size, distances were normalized by a reference, which was chosen to be the distance between the elbow and the shoulder, due to its strong correlation with body weight. To condense the information contained within these features without working with time series, we calculated the mean value, the minimum value, the maximum value, and the variance for each feature. Capturing such statistical measurements across an entire video is crucial as they collectively offer a comprehensive profile of each feature over time. The minimum and maximum values help identify the range of feature variation, while the average value provides insight into the average behavior of that feature, and the variance indicates its stability and consistency. Collectively, this approach resulted in 395 features per video (see File S1) that also included the animal sex, weight, and classical endpoints of number of foot slips and time to cross.

#### 2.5.3 Generation of Feature Sets From SimBA

To compare our custom‐engineered feature set with a more generic feature set provided by an existing “off‐the‐shelf” solution, we turned to SimBA. Videos from beam walk experiments, along with their corresponding filtered DLC tracking data, were imported into SimBA version 2.3.3 (Goodwin et al. 2024). A new pose configuration was created based on our 18 key points and used for subsequent analyses. The distances in the videos were calibrated in pixels per millimeter using the width of the 80‐cm central region of the beam as a reference value. Following the default feature extraction protocol provided by SimBA, 91 features were extracted for each video frame (see feature list in File S2). To obtain features at the single‐video level, the 91 features were averaged across the entire video, resulting in a feature set that included the x‐ and y‐coordinates for each body part and their prediction probabilities, the movement of each individual body part, and the collective movement of all body parts, along with several summary statistics on this movement across frames (sum and mean). The weight of each animal was also included as an additional feature. We refer to these features collectively in the manuscript as the “SimBA default feature set.” Additionally, we extracted an extended SimBA feature set using the “calculate feature subsets” functionality, which allowed us to extract as many additional features as possible given the custom pose configuration. This resulted in a “SimBA extended feature set” that included the default features from the default feature set, plus all pairwise distances between key points and all possible angles, totaling 4956 features (see feature list in File S3). As for the default set, animal weight was included as an additional feature, leading to a total of 4957 features used for classification. The Forestwalk workflow was then executed using either the SimBA default or SimBA extended feature set, and accuracy values were calculated for the relevant binary comparisons with the control animals for each specific experiment.

#### 2.5.4 Random Forest Classification and Feature Selection

An RFC (Breiman 2001) was utilized to explore the potential of behavioral features derived from each experiment in discriminating specific biological conditions, such as variations in genotypes or doses of a particular compound. The choice of an RFC was driven by its ability to rank features based on their importance, its ease of interpretability, robustness to overfitting due to the independent training of numerous trees, and its suitability for small input datasets, which was the case in our study. Other classifiers, such as naive Bayes (NB), k‐nearest neighbors (KNN), and support vector machine (SVM), were initially considered. However, during the initial workup of our method, we found that the RFC typically demonstrated superior performance. This could be attributed to its ability to deal with multicollinearity, which can be present in our feature set, and its capacity to capture complex feature interactions.

#### 2.5.5 Partitioning Data Into Training, Validation, and Test Sets

For each study, we employed a two‐stage leave‐one‐mouse‐out crossvalidation (LOMO‐CV) approach to evaluate the model performance and perform hyperparameter optimization. Initially, an outer LOMO‐CV was used to partition the dataset into training and test sets, where all the trials from a single animal were designated as the test set, and the remaining trials comprised the training set. Then, to optimize the model hyperparameters and perform feature selection, an inner LOMO‐CV was conducted within the training set from the outer LOMO‐CV. This involved setting aside trials from another mouse as a validation set and using the rest for training. Once the best hyperparameters were identified through the inner LOMO‐CV, they were applied to train the final model employed in the outer LOMO‐CV and obtain the final model performance. This process was iterated for each animal, ensuring that every mouse's data were used once as the test set. Note that, for the inner LOMO‐CV, the training and validation sets were used, while in the outer LOMO‐CV, the overall model performance was assessed by using the test set. Specifically, since each animal was allowed to cross each beam a maximum of two trials (as detailed in Section 2.2), the average model accuracy per animal and beam was obtained by averaging the model's accuracy for the two trials. In addition, Z‐score normalization, derived from the training set, was applied to the training set itself as well as to the validation and test sets.

#### 2.5.6 Feature Selection and Classification

Using the training and validation data in an inner LOMO‐CV fashion as described above, we (i) selected the 50 most critical features to reduce multicollinearity, increase the signal‐to‐noise ratio, and enhance the model's performance; specifically, we used the recursive feature elimination (RFE) method from the sklearn.feature_selection module for feature selection, with rfe_step = 0.05, and the RFC from the sklearn.ensemble module as the estimator, with default parameters except for class_weight = “balanced” and n_features_to_select = 50; (ii) tuned the model hyperparameters using these 50 critical features through GridSearchCV from the sklearn.model_selection module; and (iii) performed a second round of feature selection using the tuned hyperparameters and ranked all the features based on their importance, as measured by the mean decrease in impurity. Then, for every animal, we obtained a final set of prioritized features by comparing the ranked feature lists from the train/validation leave‐one‐mouse‐out split with the same test set. We created a consensus ranked list considering both the occurrence and the rank of each feature within all lists and selected the first 50. The final hyperparameters were instead obtained by taking the mode of each hyperparameter selected in all the train/validation splits. For each test set belonging to the outer LOMO‐CV, then, we trained an RFC using the specific prioritized features and hyperparameters selected from the training step, and we obtained an accuracy score ranging from 0 to 1, due to the presence of maximally two data points per animal on a specific test set.

#### 2.5.7 Linear Discriminant Analysis (LDA)

LDA was employed as a technique to visualize the separation between different biological groups within our datasets. We utilized the LDA implementation provided by the scikit‐learn library in Python (Pedregosa et al. 2011), adhering to the default parameter settings. This included using the singular value decomposition approach without assigning any priors on the class proportions. All available features in the dataset were included in the LDA. The resulting two‐dimensional projection was then used to assess and illustrate the degree of group distinction based on the given behavioral features. To further quantify the separability between the groups identified in our dataset, we measured the euclidean distance from the centroid of a reference group to each sample belonging to that group to assess the intragroup variability. Then, we compared these intragroup distances with the distances between the samples belonging to a different group and the centroid of the reference group. A larger average distance between groups, as compared to the average intragroup distances, would indicate a greater biological difference between the two groups.

#### 2.5.8 Skeleton Visualization

To visualize the average skeleton for each animal, we first standardized the x‐coordinate of the nose to zero for all animals. We then calculated the x‐coordinate for each remaining body part by using the mean distance of that body part from the nose normalized on the reference dimension. This allowed us to maintain the relative positioning of each body part while standardizing the overall skeleton size and orientation across animals. For the y‐coordinate, we used the corresponding mean y‐coordinate for each body part. This mean y‐coordinate was also used as a feature in our analysis. To represent the variance around each body part, we added bars to each body part in the visualization. These bars represent the mean variance in the vertical distance (i.e., y‐coordinate position) of each body part from the beam, as this was the primary axis of interest in our study. This method allowed us to create a clear, standardized visualization of the average skeleton for each animal, highlighting the key features and variances of interest.

### 2.6 Statistical Analysis and Study Design Factors

For pharmacological studies with diazepam, beam walk performance following vehicle administration served as the control. For studies in transgenic mice, WT littermates served as the control group. We selected a sample size based on that typically used in the literature (i.e., minimally *n* = 8 per group to provide sufficient power to detect differences of 10%–15% in group means [Carter et al. 2001]). All mice ordered were included in the study and subsequent analysis. Mice were excluded in case of a technical error (e.g., video recording failure). We set an exclusion criterion of 1 min for mice to cross the beam, although in our experience, it is rare for any mouse to exceed this threshold, and in our dataset, no mouse did so. Blinding was not performed in the study—that is, experimenters were aware of the treatment/vehicle allocation in the diazepam experiment and mouse genotype in studies with genetic models. Differences in beam‐crossing time and foot slips were analyzed using ANCOVA, with weight as a covariate for the diazepam experiment and weight and beam as covariates for other experiments in transgenic mice. Classifier accuracy against chance level (0.5) was assessed with a one‐sample Wilcoxon test. Two‐way ANOVA was applied to body weight data, considering genotype and sex as factors. Feature overlap significance was determined through bootstrap sampling (100,000 repetitions), and feature category enrichment was evaluated using the hypergeometric test.

## 3 Results

### 3.1 Forestwalk Provides Increased Sensitivity to Detect Drug Effects on Animal Posture and Balance

**FIGURE 1:**
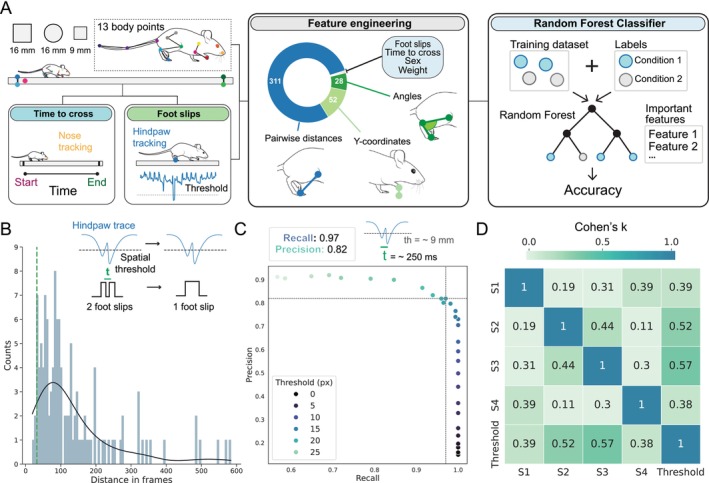
Forestwalk workflow and detection of “classical endpoints” in the beam walk test. (A) Schematic of data acquisition with DLC, and subsequent feature detection/engineering and analysis workflow (“Forestwalk”). (B) Histogram with frequency of temporal gaps (i.e., frames) between foot slips (determined by experienced human raters from 43 videos across three experiments). Green line marks lower 5% quantile; temporal cutoff below which consecutive slips are combined. Insert illustrates initial identification of slips using a y‐axis threshold, and subsequent consolidation of two events based on temporal proximity. (C) Precision‐recall curve comparing performance of the threshold‐based foot slip detection method, with varying thresholds, versus experienced human‐rater's annotations on the same dataset. Dashed lines indicate the precision (0.82) and recall (0.97) achieved using the chosen y‐axis threshold (18 pixels, ~9 mm below the beam). (D) Concordance (Cohen's kappa) between experienced human raters (“S1–S4”) and the threshold‐based detection method.

To enable automated and detailed analysis of mouse posture in the beam walk test, we first deployed DLC, an advanced tool for marker‐less pose estimation. A deep learning–based neural network was trained in DLC to label 13 distinct key points on mice as they traversed the beam (Figure 1A). These key points were selected following current state‐of‐the‐art methodologies to ensure accurate kinematic measurements of movement (Ascona et al. 2024; Weber et al., 2022) and to ensure the capture of body parts deemed important for the particular task, such as the nose to determine mouse position on the beam, the hind paw to enable automated detection of foot slips, and multiple points on the tail, which is ethologically important in the control of balance and coordination. Additionally, five points were labeled on the beams themselves, allowing automated identification of three different beams used throughout the study (i.e., 16‐mm square, 16‐mm round, and 9‐mm square beams, referred to as Beam 1, Beam 2, and Beam 3, respectively). These additional points also demarcated the central 80‐cm section of each beam where the behavioral analysis was focused (Figure 1A).

With DLC‐derived key‐point data in hand, we developed an analysis workflow for the beam walk that we refer to as “Forestwalk.” First, we aimed to automate the recording of standard metrics most often reported in the beam walk task, namely, “time to cross” and “number of foot slips.” Such metrics are typically recorded manually by human raters. However, this approach is prone to intra‐ and inter‐rater variability and likely contributes to poor reproducibility of findings from rodent behavioral neuroscience research.

The endpoint of “time to cross” was obtained simply by using the position of the animal's nose, and measuring the duration taken for the nose to enter and exit the designated central 80‐cm region of the beam (Figure 1A). Parts of the beam on either side of this region were excluded from this measure. Specifically, activity in the first 10 cm of the beam was subject to variability in how the experimenter placed the mouse at the start location, while locomotor behavior in the final 10 cm of the beam was often contaminated by exploratory activity directed toward the escape platform (e.g., pause in locomotion and stretch‐attend posture).

To enable automated detection of “number of foot slips,” we first operationally defined a foot slip as “a downward deviation of the hindpaw from its anticipated trajectory along the beam.” To verify this definition, 43 videos randomly selected from three different experimental datasets (File S4) were manually labeled for foot slips by an experienced human rater. Visual comparison of manually labeled foot slips (*n* = 162) with the hindpaw y‐coordinate confirmed that human‐labeled foot slips were consistently accompanied by a downward deviation in the trajectory of the hindpaw y‐coordinate. Moreover, this deviation appeared to exceed the typical position of the hindpaw along the beam (see example in Figure 1A). Based on this observation, we rationalized the use of a threshold‐based detection method that would count a foot slip whenever the hindpaw y‐coordinate fell below a defined spatial threshold. From the set of human‐labeled foot slips, the average time between two consecutive slip events was 138 frames, which translates to ~1 s at a recording speed of 120 frames per second, and the likelihood of two foot slips occurring within 32 frames of each other (i.e., ~250 ms) was less than 5% (Figure 1B). Thus, to avoid automated double counting of closely spaced foot slips, which were most likely part of one continuous foot slip event, a temporal threshold was also implemented to amalgamate any slip events that occurred within 32 frames of one another. With the temporal threshold fixed, systematic variation of the y‐coordinate threshold for the hindpaw position revealed that a spatial threshold of ~18 pixels below the beam surface (equivalent to ~9 mm) gave the highest level of recall (0.97) and precision (0.82) when compared to the experienced human‐rater annotations on the same set of videos (Figure 1C).

The generalizability of the threshold‐based foot slip detection method was next assessed by comparisons with four experienced human raters. The threshold‐based method produced outcomes that were consistent with those of the experienced raters and demonstrated the highest levels of agreement with most of them (Figure 1D). To ensure that our chosen threshold was not overly fitted to the initial dataset and experimental conditions, one experienced rater independently evaluated a new set of 18 videos sourced from different behavioral experiments (File S5). Foot slip events detected by the threshold‐based method were evaluated against this new, human‐rater–derived “ground truth.” The comparison again demonstrated high levels of recall (0.92) and precision (0.81), thereby validating the method's consistent performance and its capability to generalize across different experimental datasets. Collectively, these findings indicate that our threshold‐based foot slip detection method serves as a reliable and objective standard, providing a consistent measure that closely corresponds with the judgments made by multiple experienced human scorers. By contrast, agreement between different experienced human raters was generally poor (Figure 1D), which underscores the subjective nature of manual scoring and further emphasizes the value of the automated threshold–based detection method.

With the classical endpoints of “time to cross” and “number of foot slips” fully automated, we expanded the capabilities of Forestwalk to take full advantage of the 13‐labeled key points. Our expectation was that deep analysis of these data points would provide more detailed insights into postural control and balance on the beam walk than afforded by use of the classical endpoints alone.

For the next part of the Forestwalk workflow, a feature engineering step is first deployed. Our approach focused on ensuring interpretability and explainability of features, such that they could be potentially linked to underlying physiological or pathological biology and selecting features that are ethologically relevant to the beam walking task. In this context, pairwise distances between all body parts are calculated, as well as the distances from each body part to the beam surface, and essential joint angles are quantified. Each feature is initially represented as a time series, from which key statistical descriptors are extracted: the mean, minimum, maximum, and variance. This methodology, plus the inclusion of body weight, sex, and the classical endpoints of time to cross and number of foot slips, generates a dataset of 395 features for each recorded video of a mouse traversing a beam (Figure 1A). To adjust for individual differences in body size that could influence the measured features, we introduced a normalization step. Distances between body parts, as well as from each body part to the beam surface, were standardized using a reference dimension—the distance between the elbow and the shoulder. This reference dimension was selected for its consistency across subjects, allowing for more precise comparisons of postural dynamics not biased by the animal's overall body size. In addition, recognizing the significant impact of body weight on movement and balance, the weight of each animal was included as a distinct feature in the dataset, and subsequent statistical analyses included body weight as a covariate.

To refine this extensive dataset and reduce multicollinearity, a feature selection step follows in the Forestwalk workflow. RFE is deployed in tandem with an RFC to whittle down the feature set from 395 to the 50 most informative features that can effectively distinguish between experimental conditions. These 50 prioritized features are then used to train an RFC, which is designed to differentiate between experimental groups (e.g., drug treatment or genotypes). RFC performance is validated using a LOMO‐CV scheme, assessing the predictive accuracy for individual animals. This accuracy reflects the RFC's ability to correctly classify an animal into its true biological group (Figure 1A). A classifier's accuracy that significantly outperforms chance level would indicate a meaningful distinction between the conditions we aim to differentiate. When the average accuracy is found to be significantly higher than chance, it confirms that the conditions are distinct. A further benefit of the Forestwalk workflow is that prioritized features can be further analyzed to determine the most influential behavioral traits defining differences between experimental conditions (Figure 1A), thus aiding the interpretability of the results.

**FIGURE 2:**
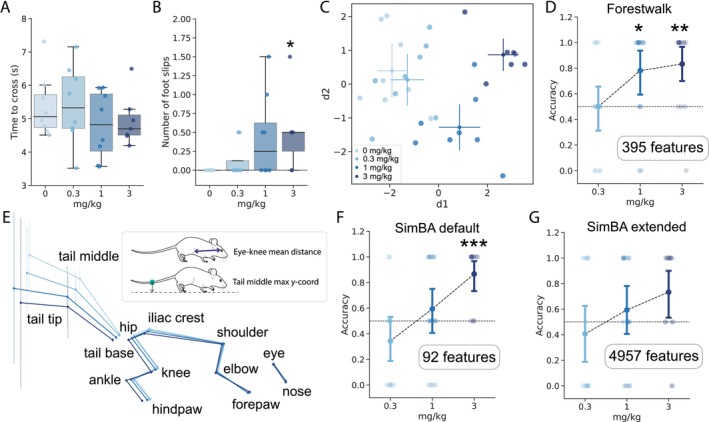
Forestwalk shows increased sensitivity to detect diazepam effects on beam walking. (A) Time to cross the beam (Beam 2) following vehicle or diazepam administration (effect of treatment; ANCOVA with weight as a covariate, *F*(3, 26) = 0.86, *p* = 0.48). (B) Foot slips on the beam (Beam 2) following vehicle (i.e., 0 mg/kg) or diazepam administration. ANCOVA with weight as covariate: *F*(3, 26) = 2.82, *p* = 0.058; post hoc analysis with Tukey's test: *p* = 0.05, 0 mg/kg versus 3 mg/kg. (C) Two‐dimensional plot with first two discriminants (d1, d2) from a linear discriminant analysis (LDA) on the diazepam dataset. (D) Point plot indicating the classifier's average accuracy to distinguish a specific diazepam treatment from vehicle. One‐sample Wilcoxon test versus chance level (0.5: dotted line): *p* = 0.023, 1 mg/kg versus chance, W = 63; *p* = 0.0063, 3 mg/kg versus chance, W = 65. (E) Skeletal representation for each group in the diazepam experiment. Points show mean position, with vertical bars showing y‐axis variance for each body part. Insert schematic highlights the two most important features that discriminate between all treatment groups. (F) The SimBA default feature set enables distinction of mice treated with 3‐mg/kg diazepam from vehicle (one‐sample Wilcoxon test vs. chance level [0.5: dotted line]: *p* = 0.001, 3 mg/kg vs. chance, W = 66). (G) The SimBA extended feature set is unable to distinguish any dose of diazepam from vehicle. Sample size *n* = 8 mice, except for 3‐mg/kg diazepam where *n* = 7. Box plots show median ± 95% CI. Two‐dimensional plot of LDA shows centroid ± 95% CI. *p < 0.05, **p < 0.01.

To evaluate the effectiveness of Forestwalk in detecting changes in beam walking performance, we conducted a pharmacological study with diazepam, a benzodiazepine known for its anxiolytic properties, but also linked to side effects of sedation and ataxia. Previous research has indicated that even low doses of diazepam can induce motor effects in humans, which are not always consistently observed in rodent models (Stanley et al. 2005). Diazepam (0.3, 1, or 3 mg/kg) or vehicle (i.e., 0 mg/kg) was administered intraperitoneally to male C57BL/6J mice, 30 min prior to starting the beam walk test, using a pseudorandomized within‐subjects crossover design (total study *n* = 8; note that at the highest dose level, one mouse failed to complete two trials on Beam 2, and thus, the final *n* for this beam is *n* = 7). The automated measures of “time to cross” did not change with any dose of diazepam in comparison to vehicle, while the “number of foot slips” significantly increased only at the highest dose of 3 mg/kg (Figure 2A,B for Beam 2, and note similar outcomes on other beams in Figure S1A–F). An LDA applied to all 395 features indicated more nuanced treatment effects of diazepam (Figure 2C). A distinct separation between animals administered with vehicle and 1 mg/kg of diazepam was already evident in LDA, which became more pronounced at 3 mg/kg, as confirmed by the increased euclidean distance from the vehicle centroid (Figures 2C, S1I). To substantiate these findings, an RFC was trained to differentiate between the vehicle control and diazepam‐treated groups. A significant difference between vehicle and 1‐mg/kg diazepam was identified, and the difference was even more pronounced between vehicle and 3‐mg/kg diazepam (Figure 2D). Taken together, these data highlight the superior sensitivity of Forestwalk to detect pharmacological effects of diazepam on beam walking performance, which were not evident from the two classical endpoints of “time to cross” and “foot slips” alone.

An average skeletal representation of mice from each treatment group was generated to further explore potential postural changes following diazepam administration (Figure 2E). A progressive elongation of the body and a lowering of the center of mass with increasing doses of diazepam were observed, along with a downward shift in tail position (Figure 2E and see an example video in File S6). In line with these observations, the mean distance from eye to knee and the maximum y‐coordinate of the tail's central point were identified by Forestwalk as the top‐ranked prioritized features in discriminating between the different diazepam doses (Figure 2E; see File S1). Thus, as the dose of diazepam increases, there is a corresponding progressive impact on the animals' posture and tail positioning. Forestwalk is sensitive to identifying these subtle but relevant changes in body posture and balance and enables their quantitative utilization for behavioral phenotyping.

To evaluate whether our customized feature set for beam walking offered any advantage over more generic key‐point–based feature sets, we performed feature extraction for the diazepam experiment using the SimBA platform (Goodwin et al. 2024). SimBA is a highly versatile and user‐friendly tool that facilitates the extraction of interpretable behavioral features from videos processed by DLC or other deep learning models that detect key points of interest. The default feature set obtained from SimBA generated 92 features per video (see Section 2.5.3 for further details). These 92 features were fed into the Forestwalk workflow to calculate the average accuracy for distinguishing animals treated with different doses of diazepam from vehicle controls. With this approach, a group difference was also identified at the 3‐mg/kg diazepam dose level (Figure 2F). However, unlike results obtained with our customized feature set, the default SimBA feature set could not discriminate between animals treated with 1‐mg/kg diazepam or vehicle (Figure 2F; and see File S2 for list of prioritized features). We repeated this analysis, but now using an extended dataset of 4957 features provided by SimBA. Despite this high number of features and being computationally intensive, the RFC was unable to distinguish between any of the diazepam dose groups and vehicle controls (Figure 2G; and see File S3 for list of prioritized features). This poor performance likely reflects the presence of many noisy or redundant features in the extended feature set. Collectively, these findings underscore the importance of using a feature set tailored to the specific task to achieve high performance. Our Forestwalk feature set, tailored specifically for beam walking analysis, demonstrates superior capability in capturing subtle behavioral differences as compared to more generic feature sets.

### 3.2 Forestwalk Reveals Previously Hidden Phenotypes in Mouse Models for AS

The beam walk test is often used to study genetic models for neurological disorders, and thus, we asked if Forestwalk could provide new insights in such a context. We focused first on mouse models for AS, a neurodevelopmental disorder marked by the absence of the maternal Ube3a protein (Jiang et al. 1999; Williams et al. 1995). Individuals with AS and mouse models used for AS research are reported to exhibit impaired movement and balance. However, the extent of motor impairments varies across different AS transgenic mouse models, and the underlying causes remain elusive (H. Huang et al. 2013; Lewis et al. 2019; Rotaru et al. 2020; Sonzogni et al. 2018; Syding et al. 2022).

**FIGURE 3:**
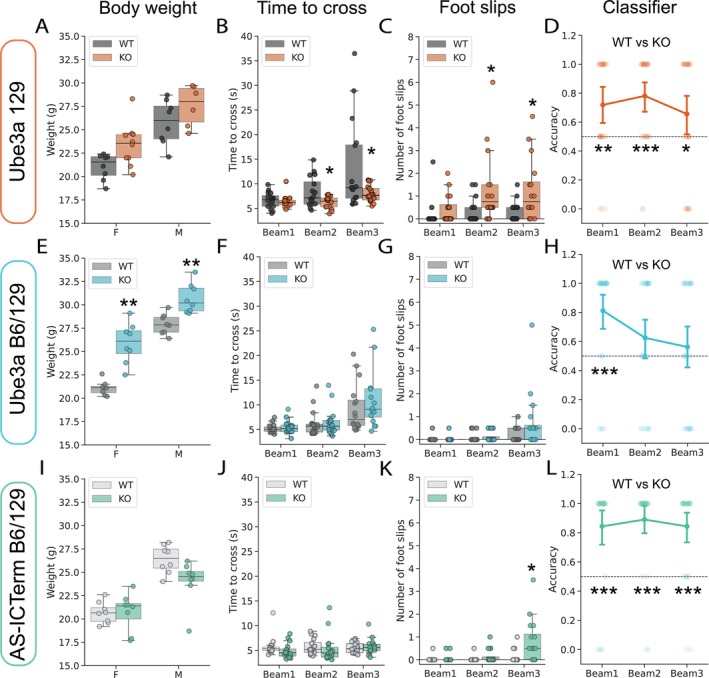
Forestwalk discriminates transgenic mouse models for Angelman syndrome in beam walking. (A) Weight distribution in female (F) and male (M) Ube3a 129 mice (KO) and wild‐type (WT) controls. Significant effect of genotype: two‐way ANOVA, *F*(1, 28) = 7.96, *p* = 0.009, and sex: *F*(1, 28) = 34.62, *p* < 0.0001. (B) Time to cross the three different beams. Significant effect of genotype: ANCOVA, *F*(1, 89) = 5.32, *p* = 0.02, beams: *F*(2, 89) = 9.08, *p* = 0.0003 and genotype * beam interaction: *F*(2, 89) = 3.1, *p* = 0.05. (C) Number of foot slips on the three different beams. Significant effect of genotype: ANCOVA, *F*(1, 89) = 8.85, *p* = 0.004. (D) Classification accuracies of WT versus KO mice per beam in the Ube3a 129 line. Dotted line indicates chance (0.5). Discrimination between genotypes was observed across all three beams. One‐sample Wilcoxon test: Beam 1, W = 147, *p* = 0.0026; Beam 2, W = 121, *p* = 0.027; Beam 3: W = 125, *p* = 0.06. (E) As for (A), but in Ube3a B6/129 mice. Significant effect of genotype: two‐way ANOVA, *F*(1, 28) = 51.56, *p* < 0.0001, and sex: two‐way ANOVA, *F*(1, 28) = 119.82, *p* < 0.0001. (F) As for (B), but in Ube3a B6/129 mice. Significant effect of beam: ANCOVA, *F*(2, 89) = 9.84, *p* = 0.0001, but no effect of genotype, and no beam * genotype interaction. (G) As for (C), but in Ube3a B6/129 mice. Significant effect of beam: ANCOVA, *F*(2, 89) = 5.62, *p* = 0.005, but no effect of genotype, and no beam * genotype interaction. (H) As for (D), but in Ube3a B6/129 mice. Discrimination between genotypes was observed in Beam 1 only. One‐sample Wilcoxon test: Beam 1, W = 290, *p* = 0.0002. (I) As for (A), but in AS‐ICTerm mice. Significant effect of sex: two‐way ANOVA, *F*(1, 28) = 49.92, *p* < 0.0001, but no genotype or sex * genotype interaction. (J) As for (B), but in AS‐ICTerm mice. No effect of beam, genotype, or beam * genotype interaction. (K) As for (C), but in AS‐ICTerm mice. Significant effect of genotype: ANCOVA, *F*(1, 89) = 6.62, *p* = 0.012, and beam: *F*(2, 89) = 9.33, *p* = 0.0002. (L) As for (D), but in AS‐ICTerm mice. Discrimination between genotypes was observed across all three beams. One‐sample Wilcoxon test: Beam 1, W = 341, *p* < 0.0001; Beam 2, W = 375, *p* < 0.0001; Beam 3: W = 297, *p* < 0.0001. Sample size = 8 mice per sex and genotype (i.e., *n* = 16 mice per genotype). Box plots show median ± 95% CI, point plots show mean ± 95% CI. ANCOVA was performed to adjust for body weight, with both beam and genotype as the factors of interest. Only significant comparisons with control animals are shown. *p < 0.05, **p < 0.01, ***p < 0.001.

We focused on three commonly used mouse models: (1) Ube3a 129 mice (129‐Ube3a^tm1Alb^/J, Jax ID: 004477), with a targeted deletion in maternal Ube3a exon 2 (Jiang et al. 1998); (2) Ube3a B6/129 hybrid mice (B6.129S7‐Ube3a^tm1Alb^/J, Jax ID: 016590), with a similar deletion but in a mixed genetic background (Jiang et al. 1998); and (3) AS‐ICTerm mice (C57BL/6J‐Rr70^em1Rsnk^/Mmmh, 065423‐MU), which have a disruption in the maternal imprinting center affecting Ube3a and several other genes (Lewis et al. 2019). All lines were bred to obtain WT littermates to serve as controls to the respective Ube3a mutant animals (commonly referred to as “KO”). Ube3a mutant mice have previously been reported as overweight compared to WT controls (H. Huang et al. 2013; Sonzogni et al. 2018). Likewise, in our study, a comparison of body weight indicated significant genotype–related differences in the Ube3a 129 (Figure 3A) and Ube3a B6/129 (Figure 3E) strains, but not in the AS‐ICTerm mice (Figure 3I).

In the beam walk test, Ube3a 129 KO mice required less time to cross the more challenging Beams 2 and 3 (Figure 3B) and performed more foot slips (Figure 3C) compared to their WT counterparts. However, further in‐depth phenotyping by Forestwalk also identified differences between KO and WT mice on the least challenging Beam 1, in addition to Beams 2 and 3, underscoring the increased sensitivity of our method (Figure 3D). Ube3a B6/129 KO mice did not exhibit significant impairments on classical beam walk endpoints compared to WT mice (Figure 3F,G). In contrast, the RFC successfully distinguished between the two genotypes on Beam 1, again highlighting behavioral differences not captured by classical endpoints alone (Figure 3H). In the case of AS‐ICTerm B6/129 KO mice, the time to cross beams was comparable to WT mice (Figure 3J), but mutant mice made more foot slips on the most challenging Beam 3 (Figure 3K). In this strain, the RFC identified significant differences between genotypes across all three beams (Figure 3L). To further probe the sensitivity of Forestwalk and our customized feature set, we repeated analysis of data from the Ube3a B6/129 mice but this time using the SimBA default feature set, which appeared most sensitive for the diazepam study (see Figure 2). Use of this more generic feature set, when deployed within the Forestwalk workflow, was unable to distinguish WT and KO mice across any of the three beams (Figure S2A; and see File S2 for list of prioritized features). Collectively, these findings demonstrate how the customized Forestwalk workflow enables the detection of differences between experimental study groups in the beam walk task that would have gone unnoticed if using classical endpoints alone.

**FIGURE 4:**
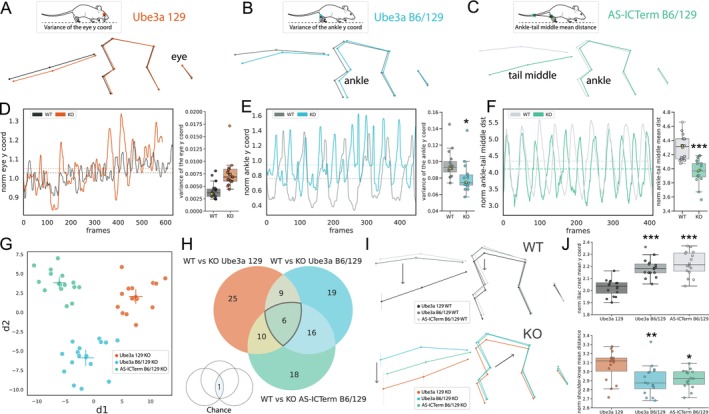
Detailed comparisons of motor coordination and balance among different transgenic mouse models for Angelman syndrome are enabled by Forestwalk. (A–C) Schematics of the average skeletal representation for each mouse line. Vertical bars show y‐axis variance for each body part. The insert on top highlights the most important features discriminating KO from WT mice in each line. (D) Traces of normalized eye y‐coordinates during a 16‐mm square beam (Beam 1) walking trial are shown for representative animals of Ube3a 129 KO (orange) and WT (dark gray) genotypes. The associated box plot shows the variance of the eye y‐coordinate. The data points of representative animals shown in the traces are highlighted in yellow. (E) As for (D), but with normalized ankle y‐coordinates for Ube3a B6/129 KO (blue) and WT (gray) genotypes. Significant difference between genotypes: ANCOVA, *F*(1, 29) = 5.55, *p* = 0.03. (F) As for (D), but with normalized ankle‐tail middle distance for AS‐ICTerm B6/129 KO (blue) and KO (gray) genotypes. Significant difference between genotypes: ANCOVA, *F*(1, 29) = 33.85, *p* < 0.0001. (G) Two‐dimensional plot reporting the first two discriminants (d1, d2) resulting from LDA on KO animals from the Ube3a 129, Ube3a B6/129, and AS‐ICTerm B6/129 mouse lines. (H) Venn diagram illustrating feature overlap among the three different transgenic mouse lines. A significant commonality of prioritized features is observed versus chance (assessed by bootstrapping with 100,000 repetitions). (I) Skeletal representations shown in (A)–(C) are replotted and grouped by either KO or WT genotype. (J) Top box plot: elevated y‐coordinate positions of the iliac crest in Ube3a B6/129 WT and AS‐ICTerm B6/129 WT mice compared to Ube3a 129 WT mice. Bottom box plot: shorter shoulder‐to‐knee distances in Ube3a B6/129 KO and AS‐ICTerm B6/129 KO mice relative to Ube3a 129 KO. Sample size = 16 mice per genotype. Box plots show median ± 95% CI. Two‐dimensional visualization of LDA shows centroid ± 95% CI. ANCOVA was performed to adjust for body weight, with genotype as the factor of interest. *p < 0.05, **p < 0.01, ***p < 0.001.

We next sought to understand which behavioral traits were responsible for genotype‐level differences detected by Forestwalk, by taking a detailed look at body posture and prioritized features from the machine learning workflow. As significant differences between WT and KO mice were detected on Beam 1 for all strains, the subsequent analysis focused on this specific beam. For Ube3a 129 mice, no differences were seen in posture or tail position in the skeletal representations of WT and KO mice in this line (Figures 4A and S3A,D). Correspondingly, the most critical features identified in Forestwalk were predominantly associated with variances in distances or angles. This suggests that the distinction between KO and WT Ube3a 129 mice lies rather in the variability of these features, but not their mean values (enrichment analysis *p* = 0.06, 41% enrichment). Indeed, this is exemplified by the variance of the eye y‐coordinate being the most significant feature identified in Forestwalk. The time‐series plot of the eye y‐coordinate in KO mice displayed more pronounced and irregular fluctuations relative to WT mice; patterns often linked to compromised balance and increased incidence of missteps (Figure 4D and see example video in File S7).

In Ube3a B6/129 mice, visual inspection of skeletal overlays suggested an alteration in body posture. Ube3a B6/129 KO mice displayed a reduced overall body length and a more compact posture compared to WT mice (Figure 4B). This observation was confirmed by a significant decrease in the average distance between the shoulder and the knee in Ube3a B6/129 KO animals compared to WT littermates (Figure S3B,E). Analysis of prioritized features from Forestwalk revealed no overall category enrichment, for example, in feature variance, distances, or mean positions, yet the variance in the ankle y‐coordinate emerged as the most significant feature (Figure 4B). Time‐series analysis of representative subjects showed that Ube3a B6/129 KO mice had smaller, yet consistent, ankle y‐coordinate fluctuations, implying a distinct and more constrained range of ankle movement relative to WT mice (Figure 4E and see example video in File S7).

Finally, AS‐ICTerm B6/129 mice exhibited alterations in body posture akin to those observed in Ube3a B6/129 mice (Figure 4C). However, in this instance, only a trend was observed toward a reduction in the distance from the shoulder and the knee, without reaching statistical significance (Figure S3C). This implies that AS‐ICTerm B6/129 KO mice may also have a tendency for a more compact posture, but the difference is less obvious than in Ube3a B6/129 mice. Notably, KO mice of this strain displayed a distinct tail posture, with the tail positioned considerably lower than WT counterparts (Figures 4C and S3F). Indeed, the most prominent feature identified in Forestwalk was the average distance between the ankle and the midpoint of the tail, which was confirmed as significantly shorter between WT and Ube3a B6/129 mutant mice (Figure 4F) and likely reflects the substantial change in the overall tail position (see example video in File S8).

With an understanding of phenotypic differences segregating KO and WT mice for individual transgenic models, we next used LDA to further explore similarities among the three sets of KO mice. The analysis along the first discriminant axis (d1) allowed us to observe a pattern of separation among the KO mice from different strains. This pattern ranged from AS‐ICTerm B6/129 KO to Ube3a B6/129 KO and then to Ube3a 129 KO, indicating that AS‐ICTerm B6/129 KO mice are phenotypically closer to Ube3a B6/129 KO mice than to Ube3a 129 KO mice (Figure 4G). On the other hand, the second discriminant axis (d2) clearly distinguished Ube3a B6/129 KO mice from both AS‐ICTerm B6/129 KO and Ube3a 129 KO mice. This separation implies that although these strains differ in some behavioral characteristics, they also share other traits that set them apart from the Ube3a B6/129 strain, highlighting the unique behavioral signature of each mouse line (Figure 4G). Consistent with this view, a comparison of prioritized features distinguishing WT from KO animals in each mouse line revealed a greater overlap between Ube3a B6/129 and AS‐ICTerm B6/129 mice (22 shared features; Figure 4H) than with Ube3a 129 mice (15 shared features with Ube3a B6/129 mice, 16 with AS‐ICTerm B6/129 mice; Figure 4H).

A comparison of prioritized features that distinguished KO from WT mice across all strains identified six that were common (Figure 4H). Notably, three of these common features were related to tail position or point variance and may reflect balance disturbances common to the different mutant strains (File S1). These shared features did not include postural‐related measures, suggesting that postural changes may be specific to the B6/129 background, rather than the genetic perturbations common to the Ube3a KO animals. Indeed, comparison of skeletal representations of all WT mice (Figure 4I) revealed similar body posture between WT mice from the Ube3a B6/126 and AS‐ICTerm B6/129 strains, which was distinct from WT Ube3a 129 mice. The higher body posture of the B6/129 strains versus Ube3a 129 WT mice was confirmed by statistical comparisons of the average y‐coordinate of the iliac crest (Figure 4J). Meanwhile, comparison of skeletal representations for all KO mice (Figure 4I) similarly revealed elevated posture in KO mice from the Ube3a B6/126 and AS‐ICTerm B6/129 strains, which was distinct from KO Ube3a 129 mice. However, KO mice from the mixed B6/129 background also displayed a more compact body posture on the beam than Ube3a 129 KO mice, which was evidenced by a shorter mean shoulder‐to‐knee distance (Figure 4I,J), and may reflect the additional impact of genetic modifiers on posture in these mice. Collectively, these findings illustrate how the Forestwalk method, utilizing intricate skeletal mappings and prioritized feature extraction, can reveal shared and strain‐unique motor deficits across different transgenic models.

### 3.3 Phenotypes Identified by Forestwalk Replicate Across Independent Experiments

A potential concern for our approach was that prioritized features and postural phenotypes identified in one experiment may not be sufficiently generalizable or reproducible between similar experiments. To empirically test the robustness and transferability of Forestwalk, we replicated our study with a new cohort of animals, specifically selecting the AS‐ICTerm B6/129 mice due to their minimal representation in prior published research.

**FIGURE 5:**
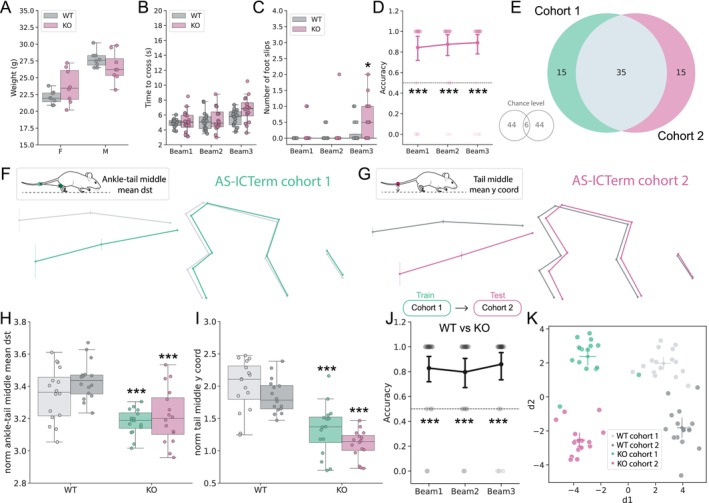
Phenotypes identified by Forestwalk are stable between independent cohorts of AS‐ICTerm B6/129 mice. (A) Weight distribution in female (F) and male (M) AS‐ICTerm B6/129 knockout (KO) and wild‐type (WT) controls from Cohort 2. Significant effect of sex: two‐way ANOVA, *F*(1, 28) = 41.36, *p* < 0.0001, and sex * genotype interaction: *F*(1, 28) = 4.6, *p* = 0.04. (B) Time to cross the three different beams. No significant effect of genotype. (C) Number of foot slips per beam. Significant effect of genotype: ANCOVA, *F*(1, 89) = 7.04, *p* = 0.009: Tukey's test: *p* = 0.029 for Beam 3 in KO versus WT mice. (D) Classification accuracies of WT versus KO mice in Cohort 2 per beam. A significant discrimination was achieved in all beams (one‐sample Wilcoxon test, Beam 1: W = 341, *p* < 0.0001; Beam 2: W = 348, *p* < 0.0001; Beam 3: W = 400, *p* < 0.0001). (E) Venn diagram showing number of prioritized features shared by AS‐ICTerm B6/129 Cohorts 1 and 2. The number of features in common was significantly different from chance, as shown in the insert (*p* < 0.0001, assessed by bootstrapping with 100,000 repetitions). (F) Visualization of skeletons per genotype for AS‐ICTerm B6/129 Cohort 1 and (G) for Cohort 2. Inserts show the most important features for the respective cohorts. (H) Comparison of distance between the ankle and the tail midpoint in both cohorts. Significant effect of genotype: ANCOVA, *F*(1, 59) = 82.64, *p* < 0.0001. Tukey's post hoc test: Cohort 1 KO versus Cohort 1 WT *p* < 0.0001 (mean difference = 0.35, 95% CI: [0.17, 0.53]), Cohort 1 WT versus Cohort 2 KO *p* < 0.0001 (mean difference = −0.43, 95% CI: [−0.61, −0.24]), Cohort 2 WT versus Cohort 2 KO *p* < 0.0001 (mean difference = −0.50, 95% CI: [−0.68, −0.32]), Cohort 2 WT versus Cohort 1 KO *p* < 0.0001 (mean difference = 0.43, 95% CI: [0.25, 0.61]). (I) Comparison of tail middle y‐coordinate in both cohorts. Significant effect of genotype: ANCOVA, *F*(1, 59) = 74.91, *p* < 0.0001 and of Cohort: ANCOVA, *F*(1, 59) = 7.8, *p* = 0.007. Tukey's post hoc test: Cohort 1 WT versus Cohort 1 KO *p* < 0.0001 (mean difference = 0.67, 95% CI: [0.37, 0.98]), Cohort 1 WT versus Cohort 2 KO *p* < 0.0001 (mean difference = −0.90, 95% CI: [−1.21, −0.60]), Cohort 2 WT versus Cohort 2 KO *p* < 0.0001 (mean difference = 0.75, 95% CI: [0.44, 1.05]), Cohort 2 WT versus Cohort 1 KO *p* < 0.0001 (mean difference = 0.52, 95% CI: [0.21, 0.83]). (J) Classifier accuracy when trained on the Cohort 1 dataset, and tested to discriminate WT versus KO mice in Cohort 2. Significant discrimination between genotypes observed in all beams. One‐sample Wilcoxon test, Beam 1: W = 294, *p* < 0.0001; Beam 2: W = 247, *p* < 0.0001; Beam 3: W = 345, *p* < 0.0001. (K) Two‐dimensional plot reporting the first two discriminants (d1, d2) resulting from LDA on AS‐ICTerm B6/129 Cohorts 1 and 2. Sample size = 8 mice per sex, 16 mice per genotype in each cohort. Box plots show median ± 95% CI. Two‐dimensional visualization from LDA shows centroid ± 95% CI. ANCOVA was performed to adjust for body weight, with genotype as the factor of interest. Only significant comparisons with control animals are shown. *p < 0.05, ***p < 0.001.

As per Cohort 1 of AS‐ICTerm B6/129 mice, no discernable differences in body weight were observed between WT and KO genotypes in the new Cohort 2 (Figure 5A cf. Figure 4I). Consistent results were also seen in the classical beam walk endpoints, with no genotype difference in time to cross the beam in Cohort 2 (Figure 5B cf. Figure 4J), and an increase in the number of foot slips by AS‐ICTerm B6/129 KO mice versus WT controls only on the most challenging Beam 3 (Figure 5C cf. Figure 3K). Likewise, the RFC effectively discriminated between KO and WT mice across all three beams (Figure 5D cf. Figure 3L). To understand if the RFC had prioritized new features to differentiate KO and WT mice in Cohort 2 than those used for Cohort 1, a comparison of prioritized features was made. Notably, prioritized features used by independent classifiers in the two experiments showed significant overlap (Figure 5E). Comparing the average skeletal positions between genotypes for both cohorts revealed consistent alterations in tail positioning and animal posture (Figure 5F,G). These observations were reflected by significant genotype differences in prioritized features used by the classifiers in both cohorts, including the “mean distance from the ankle to the tail's midpoint,” and the “mean y‐coordinate of the tail's midpoint” (Figure 5H,I). Further highlighting the robustness of prioritized features to describe the genotype‐specific behavior across different experiments, an RFC trained only on Cohort 1 could accurately distinguish KO from WT mice in Cohort 2 (Figure 5J). Finally, LDA, using all 395 engineered features from both experiments on Beam 1, revealed that the first discriminant (d1) mostly reflected differences between genotypes, while the second discriminant reflected batch effects (d2; Figure 5K). Collectively, this replication experiment identifies a stable postural trait in AS‐ICTerm B6/129 mice that could be the subject of further investigation, for example, in the context of genetic rescue experiments. Moreover, while batch effects are present within the total feature set, Forestwalk appears to not overfit the data and can be used to identify robust, generalizable, and biologically meaningful features that exhibit discriminative power between independent experiments.

### 3.4 Forestwalk Identifies Phenotypic Differences in Heterozygous (HE) and Homozygous (HO) GAT1 KO Mice

In a final assessment of Forestwalk to detect genotype/phenotype differences, we turned to the GAT1 KO mouse (B6.129S1‐Slc6a1^tm1Lst^/Mmucd; Jensen et al. 2003). This line has been used to model SLC6A1‐related neurodevelopmental disorder, which is characterized by epilepsy, intellectual disability, autism spectrum disorders, and motor impairments (Goodspeed et al. 2020). HO GAT1 KO mice show robust behavioral impairments, including hypoactivity, tremors, and coordination deficits (Chiu et al. 2005). By contrast, HE GAT1 KO mice are reported to show no overt behavioral alterations (Chiu et al. 2005), despite the presence of intermittent epileptiform activity on EEG (Lindquist et al. 2021), and the fact that human carriers of single GAT1 mutations exhibit severe symptoms (Goodspeed et al. 2023). Thus, here, we aimed to further validate the Forestwalk approach to confirm severe motor impairments in GAT1 HO mice, while also exploring its potential to reveal yet undocumented motor deficits in GAT1 HE KO mice.

**FIGURE 6:**
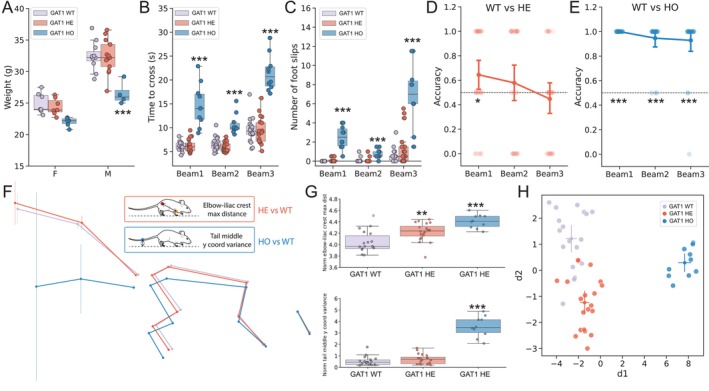
Forestwalk reveals gene‐dosage effects on beam walk posture in GAT1 knockout mice. (A) Weight distribution in female (F) and male (M) GAT1 knockout heterozygous (HE) or homozygous (HO) mice, and wild‐type (WT) littermate controls. Significant effect of genotype: two‐way ANOVA, *F*(2, 42) = 15.39, *p* < 0.0001. Tukey's test: male HO versus male WT *p* = 0.0002, male HE versus male WT *p* = 0.0002. (B) Time to cross the beam. Significant effect of genotype: ANCOVA, *F*(2, 134) = 126.92, *p* < 0.0001, and beam: ANCOVA, *F*(2, 134) = 60.20, *p* < 0.0001, and genotype * beam interaction: ANCOVA, *F*(4, 134) = 11, *p* < 0.0001. Tukey's test: GAT1 HO versus WT mice on Beam 1: *p* < 0.0001; Beam 2: *p* < 0.0001; and Beam 3: *p* < 0.0001, or GAT1 HO versus HE mice on Beam 1: *p* < 0.0001; Beam 2: *p* < 0.0001; and Beam 3: *p* < 0.0001. (C) Number of foot slips. Significant effect of genotype: ANCOVA, *F*(2, 134) = 62.15, *p* < 0.0001, of beam: ANCOVA, *F*(2, 134) = 40.36, *p* < 0.0001, and genotype * beam interaction: ANCOVA, *F*(4, 134) = 22.32, *p* < 0.0001. Tukey's test: GAT1 HO versus WT mice on Beam 1: *p* < 0.0001; Beam 2: *p* = 0.0094; and Beam 3: *p* < 0.0001, or GAT1 HO versus HE mice on Beam 1: *p* < 0.0001; Beam 2: *p* = 0.0068; and Beam 3: *p* < 0.0001. (D) Classifier accuracy in discriminating GAT1 HE from WT mice. Significant discrimination achieved in Beam 1 (one‐sample Wilcoxon test: W = 143, *p* = 0.043). (E) Classifier accuracy in discriminating GAT1 HO from WT mice. Significant discrimination achieved in all beams (one‐sample Wilcoxon test, Beam 1: W = 406, *p* < 0.0001; Beam 2, W = 325, *p* < 0.0001; Beam 3, W = 324, *p* < 0.0001). (F) Visualizations of skeletons per genotype. Inserts show the most important features in discriminating between WT and HE mice, or WT and HO mice. (G) Top box plot: maximum distance between the elbow and the iliac crest per genotype. Significant effect of genotype: ANCOVA, *F*(2, 44) = 14.03, *p* < 0.0001. Tukey's post hoc test: HE versus WT *p* = 0.004 (mean difference = −0.19, 95% CI: [−0.33, −0.05]), HO versus WT *p* < 0.0001 (mean difference = −0.38, 95% CI: [−0.55, −0.21]), HO versus HE *p* = 0.02 (mean difference = 0.19, 95% CI: [0.02, 0.35]). Bottom box plot: Variance of the y‐coordinate of the tail midpoint per genotype. Significant effect of genotype: ANCOVA, *F*(2, 44) = 76.52, *p* < 0.0001. Tukey's post hoc test: HO versus WT *p* < 0.0001 (mean difference = −3.01, 95% CI: [−3.53, −2.48]), HO versus HE *p* < 0.0001 (mean difference = 2.83, 95% CI: [2.31, 3.34]). (H) Two‐dimensional plot reporting the first two discriminants (d1, d2) resulting from LDA on GAT1 mice. Sample size = 18 WT (7 females, 11 males), 20 (7 females, 13 males) HE, and 10 HO (6 females, 4 males). Box plots show median ± 95% CI. Two‐dimensional visualization from LDA shows centroid ± 95% CI. ANCOVA was performed to adjust for body weight, with genotype as the factor of interest. Only significant comparisons with control animals are shown. **p < 0.01, ***p < 0.001.

As previously reported (Chiu et al. 2005), GAT1 HO mice had significantly lower body weight than either WT or HE animals (Figure 6A). For classical beam walk endpoints, a significant increase in the time to cross the beam and in the number of foot slips was seen in HO animals versus WT and HE mice across all beams (Figure 6B,C). As also expected from prior literature (Chiu et al. 2005), GAT1 HE KO mice showed no obvious difference in these classical endpoints as compared to WT mice (Figure 6B,C). The RFC could well discriminate between HO and WT mice across all three beams (Figure 6E), further corroborating the strong motor phenotype reported for HO GAT1 KO mice. Surprisingly, the RFC also achieved significant discrimination between WT and HE mice in Beam 1 (Figure 6D), suggesting that motor differences are present in HE GAT1 KO mice under specific test conditions, but that classical endpoints are not sensitive enough to reveal them. In addition, we investigated whether a similar distinction between WT and HE mice could be detected using the SimBA default feature set in place of our customized Forestwalk feature set. However, the use of this feature set was unable to achieve discrimination between GAT1 WT and HE across any beam (SimBA default feature set; Figure S2B and see File S2 for list of prioritized features). Thus, our customized Forestwalk feature set appears superior for identifying subtle behavioral differences between groups in beam walking.

We explored postural differences in GAT1 mutant mice in more detail and focused on Beam 1 where both HO and HE mice were discriminated from WT controls by the RFCs. The averaged skeletal alignments and prioritized features revealed substantial changes in GAT1 HO mice versus WT controls. In particular, HO mice assumed an extended posture (Figure 6F), evidenced by an increased elbow‐to‐iliac crest distance (Figures 6G and S4A). HO mice also displayed a lower center of gravity, with a reduced iliac crest height (Figure S4B) and lower hind paw position on the beam's edge (Figure S4C), likely to achieve increased stability while traversing the beam (Figure 6F and see example video in File S9). These postural changes were accompanied by extensive movements in the tail of GAT1 HO mice. Indeed, variance in the tail middle point was a top‐ranked prioritized feature distinguishing WT and HO mice (Figure 6G). Similarly, GAT1 HE mice also showed a modest extension in body length when compared to WT mice (Figures 6F,G and S4A), but this did not coincide with a significant change in the center of mass (Figure S4B). The observed elongation in posture was reflected in the prioritized features, such as the maximum distance between the iliac crest and the elbow (Figure 6G). Finally, LDA visualization further substantiated differences among genotypes. The first discriminant (d1) effectively separated HO mice, while the second discriminant (d2) distinguished HE mice from WT mice, confirming the presence of a balance phenotype in these animals (Figures 6H and S4D). Collectively, these findings underscore the ability of Forestwalk to identify genotype/phenotype relationships in motor function and open the door for further study of brain–behavior relationships in such genetic models of human disease.

## 4 Discussion

Investigating motor function through animal models is essential to understand brain function in health and disease and to develop potential therapeutic strategies (Brooks and Dunnett 2009). In this context, behavioral tests such as the beam walk provide an invaluable, controlled, and quantifiable way to evaluate coordination and balance in rodents, and also humans (Carter et al. 2001; Chaumeil et al. 2024; Modi et al. 2024). However, classical endpoints used in the rodent beam walk, including the number of foot slips and time taken to cross the beam, do not fully capture the repertoire of behaviors observed during the task. Additionally, manual scoring of classical endpoints gives rise to high variability between different observers, which limits the reproducibility of findings. Here, using machine learning–based data capture and analytical tools, we have revisited the rodent beam walk to ask what additional information can be obtained from this ethologically relevant test. We developed a new analysis workflow for the beam walk, named Forestwalk, and were able to identify previously hidden effects of pharmacological treatment and unreported phenotypes in different mouse models used to research neurodevelopmental disorders. Taken together, this new approach can advance research into brain mechanisms controlling posture and balance.

An initial objective of our study was to automate the detection of classical endpoints used in the beam walk, namely, the number of foot slips and time to cross the beam. This was achieved with Forestwalk and thus ensures a standardized and reproducible scoring approach, as compared to human raters who are prone to inter‐ and intra‐rater variability. While there is an upfront time investment required to establish Forestwalk, there is a considerable time saving in comparison to manual scoring that accumulates with each subsequent experiment. Training the deep learning model to achieve automatic tracking of key points is a necessary first step and took approximately 21 h in our hands. However, the user only needs to initiate the training process rather than actively manage it and thus can engage in other tasks while computations are taking place. Unlike “hands‐on” training that must take place for every new human scorer that joins a lab, the time‐consuming step of establishing a deep learning model is required only once. The same model can then be applied to all newly acquired experiments, significantly enhancing analysis efficiency, speed, and reproducibility as the number of experiments increases. Automated scoring of foot slips and time to cross of > 180 videos from one experiment, with a total recording duration of ~66 min, took only ~4 min when using a high‐performance computing cluster. In contrast, human raters typically score videos at a reduced playback speed or even frame by frame, so would require considerably more time to score the same data set. Running the entire Forestwalk workflow on the same experiment took ~65 min, and given the high number of features explored in each video, manual annotation and analysis of such an extensive feature set are unlikely to be even attempted by a human rater.

A further objective of our study was to improve the sensitivity of the beam walk to reveal differences between experimental groups. In one example using diazepam, we identified treatment effects with a low dose of diazepam in the expanded feature set of Forestwalk that were not seen when using classical endpoints alone. In pharmaceutical drug discovery, obtaining such insights is critical. Accurate dose–response relationships are necessary to prioritize compounds for further development, to establish the therapeutic window between efficacy and safety, and to inform dose selection for future clinical studies. Moreover, studies using beam walking typically claim successful rescue of model phenotypes when foot slips are monitored and recovered to control levels or claim the absence of a motor phenotype or treatment effect when no difference in foot slips occurs. Our study clearly demonstrates that subtle but relevant changes in posture and balance can still occur even in the absence of changes in foot slips. Thus, caution should be taken when using classical endpoints alone in drawing conclusions from the beam walk test. It should also be noted that the enhanced sensitivity observed in our Forestwalk workflow is attributed to our expansion beyond classical endpoints to include a comprehensive set of metrics describing animal movement and posture, as well as the use of an RFC to interpret these metrics collectively rather than independently. Among many studies that deploy marker‐less pose estimation for advanced behavioral analysis, there are relatively few examples of where feature interactions are taken into account in the analysis workflow (e.g., R. Huang et al. 2021), and a majority of studies tend to focus on the analysis of independent features (e.g., Bidgood et al. 2024; Piotrowski et al. 2024). Random forests, as used in our study, are particularly effective at capturing complex interactions between features, and this capability allows for a more comprehensive understanding of the data by considering how feature combinations influence outcomes.

A final objective for our research was to address the limited specificity of the beam walk task to discriminate fundamentally different experimental conditions. We focused on comparing transgenic mouse strains used to study AS, which is a genetic disorder affecting the nervous system and is characterized by developmental delay, intellectual disability, profound speech impairment, and problems with movement and balance (Angelman 1965; Williams et al. 1995). The rotarod is most often used to study balance and coordination in mouse models for AS and has identified impaired performance across several transgenic lines (H. Huang et al. 2013; Leach and Crawley 2018; Sonzogni et al. 2018) and been used to assess therapeutic interventions (Clarke et al. 2024; Cruz et al. 2021; Egawa et al. 2012; Milazzo et al. 2021; Schultz and Crawley 2020). A particular strength of the beam walk in comparison to the rotarod is that it enables an assessment of *how* the animal performs the test, not only *if* the animal performs it (Stanley et al. 2005). Such qualitative differences in performance, which can now be reliably quantified in the Forestwalk workflow, are likely to facilitate a more precise understanding of mechanisms linked to balance control in AS and would provide a more granular view on therapeutic efficacy. Indeed, we were able to identify common and unique postural traits associated with each transgenic line under investigation, which may speak to the genetic commonalities (e.g., loss of ube3a) and differences (e.g., background strains) among the lines. Similarities were identified between Ube3a B6/129 and AS‐ICTerm mice, which may reflect their shared genetic background. Across all three transgenic lines, six shared features were identified, of which three were related to tail position or movement, consistent with balance disturbances. Moreover, prioritized features distinguishing transgenic mice from controls were stable between independent cohorts of AS‐ICTerm B6/129 mice, suggesting that they could serve as defined endophenotypes of the line. In light of our findings, we suggest that further attention should be given to the beam walk in the characterization of posture and balance disturbances in rodent lines used for AS research and for future therapeutic testing.

A concern for machine learning models such as those used in our study is that they may have limited generalizability beyond the dataset and conditions on which they were initially developed. Although our DLC model was trained to detect key points on mice of different ages, weights, and fur color, other variables such as room lighting conditions, the camera used, camera positioning, and the beam equipment itself could impact on the accuracy and transferability of the model (von Ziegler et al. 2021). To overcome this, establishing a new DLC key‐point model or retraining the model used in our study with examples provided from a new experimental context is highly recommended. We further explored the generalizability of our approach by performing a replication experiment with AS‐ICTerm B6/129 mice and found that a classifier trained on one cohort of animals could well predict the genotype of mice in a second fully independent cohort. Thus, this cross‐cohort validation suggests that our model has not been overfitted to the initial group and has indeed captured essential features that differentiate the genotypes. Interestingly, when looking at the entire feature set from these experiments, we found that its information content not only pertained to the primary experimental factors under investigation (i.e., WT vs. transgenic mouse differences) but also identified global batch differences (i.e., Cohort 1 vs. Cohort 2). While potential sources of variability (e.g., beam type, time of day, room lighting conditions) were controlled between cohorts, it is clear that other factors that may contribute to between‐batch variability are less well understood (e.g., slow oscillatory activity in laboratory housed mice; Pernold et al. 2021). We suggest that the Forestwalk approach, given its improved sensitivity, can also be used to further study the influence of such variables on experimental outcomes.

To further understand if the sensitivity of Forestwalk was appropriately calibrated, we conducted studies in GAT1 KO mice (Jensen et al. 2003), a useful model for the study of SLC6A1‐related neurodevelopmental disorder (Goodspeed et al. 2020, 2023). HO GAT1 KO mice exhibit a range of motor disorders, including gait abnormalities, constant tremor, reduced rotarod performance, and reduced locomotor activity in the homecage (Chiu et al. 2005). By contrast, extensive study of HE GAT1 KO mice has failed to reveal pronounced behavioral phenotypes (Chiu et al. 2005), despite the fact that HE mice have only intermediate GABA uptake capacity (Chiu et al. 2005) and exhibit frequent spike‐wave discharges measured by electrocorticography (Lindquist et al. 2021). Consistent with published literature, we identified pronounced differences in GAT1 HO versus WT controls with both classical endpoints and using the expanded feature set of Forestwalk. For GAT1 HE mice, classical endpoints did not differ from WT controls. Nevertheless, the classifier was able to successfully categorize GAT1 HE mice in the first beam. The absence of detectable differences in GAT1 HE mice on other beams is an interesting result that requires further study but could implicate a learning acquisition effect that only manifested during exposure to the first beam in the test order and was quickly overcome. Interestingly, a genotype difference only on the first and easiest beam was also revealed in the experiment with Ube3a B6/129 mice. Another study using a touchscreen‐based test of cognition identified a robust learning acquisition deficit in Ube3a B6/129 mice (Leach and Crawley 2018), which might be relevant to our observation. To the best of our knowledge, tests of cognitive capabilities in GAT1 HE mice have not been made using highly sensitive paradigms such as the touchscreen but would be of interest in future studies. Taken together, experiments with GAT1 KO mice suggest that Forestwalk generates results that are consistent with other published reports but can yet reveal subtle group differences that may have escaped detection in other measures of motor function and warrant further exploration.

Optimizing beam walk data capture and analysis has also been the focus of other groups (Bidgood et al. 2024; Ito et al. 2022; Lang et al. 2020; Semler et al. 2011; Wan et al. 2023). For example, Ito et al. (2022) developed a “simple scoring system” with binary judgments of values, such as retention, moving forward, and reaching the goal to rate beam walking performance. A low score thus reflected poor performance, and the approach was validated using a model of SCI with comparison to another commonly used scale in the field, namely, the Basso Mouse Scale for locomotion (Basso et al. 2006). Other groups have adopted a single‐frame motion analysis (SFMA) approach in both mice (Apostolova et al. 2006; Irintchev et al. 2005) and rats (Semler et al. 2011) to assess gait deficits after spinal cord or nerve injury. In SFMA, objective parameters are obtained from individual video frames, such as the foot‐stepping angle, and these parameters were found to correlate with underlying lesion volume and to track lesion severity (Semler et al. 2011). While the simple scoring method and SFMA are both straightforward to implement, require only cheap equipment, and no specific knowledge or training of personnel (Ito et al. 2022; Semler et al. 2011), they do rely on human raters and require significant time to score or manually annotate videos.

Like in our study, other groups have turned to DLC‐based tracking to overcome the limitations of human‐rater scoring of beam walking performance (Bidgood et al. 2024; Lang et al. 2020; Wan et al. 2023). Lang et al. (2020) established a parameter to assess the rhythmicity of gait and reported changes in beam walking performance in a mouse model for spinocerebellar ataxia that were not observed in less complex studies of natural gait. Meanwhile, Wan et al. (2023) deployed FluoRender architecture to extract a standard walk cycle from 2D and 3D pose estimation and demonstrated the feasibility of the method in two mice. Most recently, Bidgood et al. (2024) combined DLC tracking with an earlier version of SimBA (Nilsson et al. 2020) and identified differences in global walking dynamics in a mouse model used for Parkinson's disease research. While SimBA focuses on behavioral classification at the single‐frame level, there also exist unsupervised methods, like keypoint‐Moseq (Weinreb et al. 2024) and behavioral flow (von Ziegler et al. 2023), which allow for clustering of behaviors at the single‐video level. However, these methods have not yet been applied in the context of beam walking. While each of these methods has its own advantages, to the best of our knowledge, Forestwalk is the only approach thus far to automate the detection of foot slips and is scalable to full experimental studies. Moreover, DLC‐labeled key points can be used in Forestwalk to reconstruct faithful skeletal representations of mice and combine engineered features with machine learning tools. Collectively, Forestwalk permits an automated and detailed exploration of determinant postural differences in mice traversing the beam that has not, to the best of our knowledge, previously been accomplished.

We generated a custom set of 395 engineered features for the Forestwalk workflow. The use of this custom feature set appeared more sensitive to detect group differences than two generic feature sets provided by the popular open‐source tool, SimBA. Thus, we believe that there is an advantage of incorporating a priori test‐specific knowledge to fully capitalize on modern machine learning tools for behavioral analysis. However, reliance on prior knowledge and assumptions can introduce bias and constrain the discovery of novel insights. To limit human bias in feature selection, we deployed an RFE step to reduce 395 features to only those that mattered most to the classification task. An alternative approach is to use data‐driven methods, like neural network–based feature extraction, that offer greater flexibility and can dynamically adjust to data (e.g., Ryait et al. 2019). However, neural networks typically function as a “black box,” making it difficult to interpret which features are driving the discrimination between groups. Indeed, there are examples of studies that set out with neural network–based feature extraction but eventually return to engineered features to support the explainability of group differences (Torabi et al. 2021). Large datasets are also needed to train neural networks for reliable feature extraction, and this can be a significant limitation when resources are constrained. In such situations, engineered features offer a more resource‐efficient way to obtain relevant and interpretable information. These considerations led us to favor engineered features to ensure interpretability and explainability of results, enabling the identification of key features that can be verified in video recordings or skeletal representations, and allowing us to more readily track phenotype progression and drug effects. Our approach also allowed us to automate the detection of classical endpoints in a straightforward way, for example, by measuring the hind paw's distance from the beam, which mirrors the approach humans use to identify foot slips. As our studies show, the Forestwalk feature set has shown remarkable sensitivity and the ability to generalize effectively across various experiments and provides new insights in the context of beam walking that have not been described previously.

The beam walk task is of particular interest given its direct translational application to human studies. Balance is important for many aspects of daily life of humans, including walking (Silverman and Neptune 2011), standing from a chair (Fujimoto and Chou 2012), or climbing stairs (Pickle et al. 2014). Impaired balance and gait, potentially leading to falls, is a major determinant of poor quality of life, immobilization and reduced life expectancy in older adults, and in several neurological conditions (Dyer et al. 2020; Fasano et al. 2017). As in our report, studies of balance in humans using beam walking are also exploring the potential for marker‐less–based motion capture to overcome issues associated with maker‐based systems (Chaumeil et al. 2024). As marker‐less pose estimation becomes more commonplace, it will be of great value to align and potentially harmonize analytical methods used in nonclinical and clinical settings where possible to further aid translational research.

## 5 Limitations

There are several limitations to our study. First, we chose a single side‐view camera, which precluded monitoring of both hind paws for foot slip detection. Our method could be improved by the use of an additional camera, or by the placement of a mirror to obtain additional views of the mouse, as recently described in a study of stroke recovery using the ladder rung test (Weber et al. 2022). Second, human studies with marker‐less–based motion capture have placed emphasis on comparisons to marker‐based methods for validation and comparison of the accuracy of these approaches (Chaumeil et al. 2024). We did not benchmark against marker‐based methods, which place an additional welfare burden in mice. Thus, it is possible that potential bias was introduced into our analysis workflow from key‐point tracking data that is difficult to objectively quantify. Third, our approach focused on mice, but the beam walk is also commonly used with rats (Goldstein and Davis 1990; Piot‐Grosjean et al. 2001; Semler et al. 2011). It would be of value in the future to extend the Forestwalk workflow to this species. We expect that a new key‐point model would need to be trained specifically for rats, and automated foot‐slip detection would need to be re‐established using different threshold parameters. However, general principles of the Forestwalk workflow would also be applicable to data obtained from studies in rats. Fourth, our approach does require computational resources and programming expertise that may not be available to all laboratories. Notwithstanding, we do believe that the additional insights gained from our approach far outweigh the investment necessary to obtain these insights, both from a scientific and ethical standpoint. Fifth, we recognize that automated analysis can contribute to reducing variability and increase experimental reproducibility. However, additional factors still need to be considered (such as the equipment type, batch effects, and learning effects due to procedural differences between laboratories) to ensure reliable and reproducible results from the beam walk that are beyond the scope of our work. Finally, here, we focused only on the beam walk and were unable to make direct comparison between performance in this task and other measures of balance and coordination, such as rotarod performance. Eventually, all tests for motor behavior have advantages and limitations, and it is typically recommended that a battery of measures appropriate for a particular study are deployed (Modi et al. 2024; Semler et al. 2011).

## 6 Conclusion

Here, we established a new analysis workflow for beam walking in mice, named Forestwalk, that automates the detection of classical endpoints used in the task and delivers more sensitive and specific insights into postural control and balance. As demonstrated by our experiments with diazepam and different transgenic strains used in the study of neurodevelopmental disorders, Forestwalk represents the current state‐of‐the‐art analysis workflow for beam walking in mice and opens the door to a better understanding of how the brain controls movement in health and disease.

## Author Contributions

**Francesca Tozzi:** data curation, formal analysis, investigation, methodology, software, validation, visualization, writing – original draft, writing – review and editing. **Yan‐Ping Zhang:** conceptualization, data curation, software, writing – original draft, writing – review and editing. **Ramanathan Narayanan:** conceptualization, supervision, writing – original draft, writing – review and editing. **Damian Roqueiro:** conceptualization, data curation, formal analysis, software, supervision, writing – original draft, writing – review and editing. **Eoin C. O'Connor:** conceptualization, funding acquisition, methodology, project administration, resources, supervision, visualization, writing – original draft, writing – review and editing.

## Conflicts of Interest

All authors were employees of F. Hoffmann‐La Roche AG, Switzerland, at the time of study conduct and original manuscript submission.

### Peer Review

The peer review history for this article is available at https://www.webofscience.com/api/gateway/wos/peer‐review/10.1111/ejn.70033.

## Supporting information


**Data S1.** Supporting Information.

## Data Availability

DLC annotated key‐point data from > 1200 video files recorded for this publication are made available at https://zenodo.org/records/14639755. Data files are provided with a Minimal Metadata Set (Moresis et al. 2024) to enable their future reuse and repurposing. Open‐source code for Forestwalk is available for download at https://github.com/Roche/neuro‐forestwalk.
